# Transition processes of a static multilevel atom in the cosmic string spacetime with a conducting plane boundary

**DOI:** 10.1038/s41598-018-30260-9

**Published:** 2018-08-07

**Authors:** Huabing Cai, Zhongzhou Ren

**Affiliations:** 1 Department of Physics, Nanjing University, Nanjing, 210093 China; 2 School of Physics Science and Engineering, Tongji University, Shanghai, 200092 China

## Abstract

We investigate the transition processes of a static multilevel atom in interaction with a fluctuating vacuum quantum electromagnetic field in the cosmic string spacetime in the presence of an infinite, perfectly conducting plane. Using the formalism proposed by DDC, we find that the presence of the boundary modifies both vacuum fluctuations and radiation reaction contributions to the atomic spontaneous emission rate. Our results indicate that the total decay rate and the boundary-induced contribution both depend upon the atom-string distance, the atom-plate separation, the extent of the polar angle deficit induced by the string, and the atomic polarization direction. By adjusting these parameters, the atomic decay rate can be either enhanced or weakened significantly by the boundary. Moreover, the presence of the boundary can distinguish certain polarization directions that bring about the same decay rate in the case of a free cosmic string spacetime. Theoretically, our work suggests a more flexible means to adjust and control the radiative processes of atoms.

## Introduction

Spontaneous emission process of atoms is one of the prominent quantum phenomena and it actually involves the interaction of atoms with the surrounding radiation field. A lot of previous works have been carried out in order to reveal its underlying mechanism, and it has been found that the quantum process can be attributable to the action of vacuum fluctuations or radiation reaction^1–6^. Thereinto, Milonni believed that the two contributions can be distinguished by choosing a particular ordering between the atomic and field operators and to a large extent, this choice is arbitrary^7^. Further, as discussed by Dalibard, Dupont-Roc and Cohen-Tannoudji (DDC), separating the field operator into the “free” part, i.e. the free field operator in the absence of the atom-field coupling, and the “source” part only caused by the atom-field coupling and choosing a symmetric atom-field operator ordering, vacuum fluctuations (“free” field) and radiation reaction (“source” field) contributions can be identified as both contributions are Hermitian and thus have independent physical meanings^8,9^. Within such a formalism, the stability of an inertial atom in the ground state in the Minkowski spacetime can be maintained by the interplay between the two contributions. Later, this formalism was widely utilized to investigate the radiative properties of an inertial or accelerated atom in interaction with a variety of quantum fields in an unbounded flat spacetime^10–14^. Recently, these surveys have been popularized to the curved spacetime background^15–21^. It is worth mentioning that those surveys for atoms in the accelerated or curved background have theoretically predicted the spontaneous excitation process.

Cosmic strings are a type of topological defects which may have been formed during a symmetry breaking phase transition of the very early universe, others such as domain walls, monopoles and textures^22^. Initially, due to their unusual properties and potential implications in cosmology^22–26^, topological defects arose considerable interest, especially cosmic strings. Particularly, the gravitational field of a simplest idealized cosmic string, namely a static, straight infinitely long and thin cosmic string, can be represented by a conical geometry and the surrounding spacetime is locally flat but has a curvature singularity at the string^27^. It has been manifested that the nontrivial spacetime structure due to the string can give rise to several remarkable physical effects, for example, electron-positron pair production by a high energy photon^28,29^, bremsstrahlung process of a freely moving charged particle^30,31^, self-force on a static electrically charged particle^32^, gravitational lensing effect^33^ and gravitational Aharonov-Bohm effect^34–36^, etc. Furthermore, in order to fully uncover the properties of the string, some important quantum processes that were explicitly investigated in the Minkowski spacetime have been increasingly extended to the case in the presence of the string, e.g., vacuum polarization^37–39^, Landau quantization^40^, Berry phase^41,42^, the Casimir effect^43,44^ and the Casimir-Polder interaction^45–47^. In the respect of the atomic radiative properties, let us list some previous works. As a toy model, the radiative properties of a static two-level atom immersed in a thermal bath of a massless scalar quantum field were investigated in a cosmic string spacetime^48^ or a global monopole spacetime^20^, and it was found that the atomic transition rates rest with the distance of the atom with the topological defect. In a more realistic situation, the radiative processes of a static multilevel atom^49^ or two two-level entangled atoms^50^ in interaction with a quantum electromagnetic field were investigated in the presence of a cosmic string. Notably, their results indicate that the atomic transition rates are crucially dependent on the atomic polarizability with respect to the string, in addition to the distance of the atom with the string. Besides, the combined effects of the acceleration and the topological defect on the transition processes of atoms were also investigated^51^.

According to the quantum field theory, we know that the presence of boundaries in a Minkowski spacetime can modify the eigen modes of quantum fields and accordingly affects the quantum fluctuations. The influence in quantum fluctuations will give rise to many novel observable phenomena, such as the Casimir effect^52,53^ and the Casimir-Polder interaction^54–61^. On the other hand, it has been manifested that the radiative properties of atoms can be influenced by the existence of boundaries^62–70^. So in this paper, we are interested to see what happens to the transition processes of a static atom in the background of a cosmic string when there exists a conducting plane boundary, as the case of a free cosmic string spacetime has been investigated clearly^49^. In principle, the presence of the boundary can modify the quantum fluctuations of electromagnetic field in cosmic string spacetime and accordingly influences the transition processes of atoms via the atom-field coupling. Using the formalism proposed by DDC, we separate the contributions of vacuum fluctuations and radiation reaction to the atomic transition rates and explore how they rely on the boundary. Through the comparison of our results with that of a free cosmic string spacetime^49^, we can reveal the modifying effects of the boundary. It should be noted that we adopt the natural units *ħ* = *c* = 1 throughout the paper.

## Methods

### Interaction of a multilevel atom with a fluctuating electromagnetic field in the background of a cosmic string

The spacetime around an idealized cosmic string can be described by the line element^27^

1 $$d{s}^{2}=d{t}^{2}-d{\rho }^{2}-{\rho }^{2}d{\theta }^{2}-d{z}^{2},$$

where *ρ*, *θ* and *z* are the cylindrical coordinates and 0 ≤ *θ* < 2*π*/*ν*. The parameter *ν* is linked to the string’s linear mass density *μ* by *ν* = (1−4 *Gμ*)^−1^, where *G* is the Newton’s constant. In comparison to the case of the Minkowski spacetime, the above line element apparently describes a locally flat and cylindrically symmetric spacetime, characterized by a polar angle deficit 8 *πGμ*. Now, in this spacetime context, let us consider the interaction of a multilevel atom with a quantum electromagnetic field. We choose to work in the proper reference frame of the atom, so the evolution of the whole system with regard to the atomic proper time *τ* is governed by the total Hamiltonian *H*(*τ*) = *H*_*A*_(*τ*) + *H*_*F*_(*τ*) + *H*_*I*_(*τ*). Thereinto, the Hamiltonian operator of the multilevel atom, *H*_*A*_, is expressed as

2 $${H}_{A}(\tau )=\sum _{n}{\omega }_{n}{\sigma }_{nn}(\tau ),$$

in which *σ*_*nn*_ = |*n*〉〈*n*|, *n* labels a complete set of stationary atomic states and *ω*_*n*_ denotes the corresponding energies. *H*_*F*_(*τ*) is the Hamiltonian operator of the quantum electromagnetic field,

3 $${H}_{F}(\tau )=\sum _{{\boldsymbol{k}}\lambda }{\omega }_{{\boldsymbol{k}}}{a}_{{\boldsymbol{k}}\lambda }^{\dagger }{a}_{{\boldsymbol{k}}\lambda }\frac{dt}{d\tau },$$

where *a*_***k****λ*_$({a}_{{\boldsymbol{k}}\lambda }^{\dagger })$ denotes the annihilation (creation) operator for a photon with the wave vector ***k*** and the polarization *λ*, and *ω*_***k***_ gives the corresponding energy. The Hamiltonian operator *H*_*I*_(*τ*) should describe the atom-field interaction, and in the multipolar coupling scheme^71,72^ we introduce the form

4 $${H}_{I}(\tau )=-\,e{\bf{r}}(\tau )\cdot {\bf{E}}(x(\tau )),$$

where *e* is the charge of an electron, *e***r** is the atomic electric dipole moment operator, **E**(*x*) denotes the electric field operator and *x*(*τ*) the atomic trajectory.

### General expression of the rate of change of the atomic energy

As we are interested in the radiative processes of the atom, we now write out, in the Heisenberg picture, the evolution equation for the atomic Hamiltonian,

5 $$\frac{d{H}_{A}(\tau )}{d\tau }=-\,ie[{{\rm{r}}}_{i}(\tau ){{\rm{E}}}_{i}(x(\tau )),{H}_{A}(\tau )].$$

As the DDC formalism have been employed in some previous works^10–21^, we immediately obtain the general expression of vacuum fluctuations and radiation reaction contributions to the rate of change of the atomic energy,

6 $${\langle \frac{d{H}_{A}(\tau )}{d\tau }\rangle }_{{\rm{v}}{\rm{f}}}=2i{e}^{2}{\int }_{{\tau }_{0}}^{\tau }d\tau ^{\prime} {C}_{ij}^{F}(x(\tau ),x(\tau ^{\prime} ))\frac{d}{d\tau }{({\chi }_{ij}^{A})}_{b}(\tau ,\tau ^{\prime} ),$$

7 $${\langle \frac{d{H}_{A}(\tau )}{d\tau }\rangle }_{{\rm{r}}{\rm{r}}}=2i{e}^{2}{\int }_{{\tau }_{0}}^{\tau }d\tau ^{\prime} {\chi }_{ij}^{F}(x(\tau ),x(\tau ^{\prime} ))\frac{d}{d\tau }{({C}_{ij}^{A})}_{b}(\tau ,\tau ^{\prime} ),$$

where 〈⋅⋅⋅〉 = 〈0, *b*|⋅⋅⋅|0, *b*〉. In fact, the above expressions are obtained in a perturbation treatment to order *e*^2^ and we have assumed that the field is initially in the vacuum state |0〉 and the atom is prepared in an arbitrary stationary state |*b*〉. Thereinto, ${C}_{ij}^{F}(x(\tau ),x(\tau ^{\prime} ))$ and ${\chi }_{ij}^{F}(x(\tau ),x(\tau ^{\prime} ))$ are, respectively, the symmetric correlation function and the linear susceptibility of the electromagnetic field along the atomic trajectory, defined as

8 $${C}_{ij}^{F}(x(\tau ),x(\tau ^{\prime} ))=\frac{1}{2}\langle 0|\{{{\rm{E}}}_{i}^{f}(x(\tau )),{{\rm{E}}}_{j}^{f}(x(\tau ^{\prime} ))\}|0\rangle ,$$

9 $${\chi \,}_{ij}^{F}(x(\tau ),x(\tau ^{\prime} ))=\frac{1}{2}\langle 0|[{{\rm{E}}}_{i}^{f}(x(\tau )),{{\rm{E}}}_{j}^{f}(x(\tau ^{\prime} ))]|0\rangle .$$

Similarly, ${({C}_{ij}^{A})}_{b}(\tau ,\tau ^{\prime} )$ and ${({\chi }_{ij}^{A})}_{b}(\tau ,\tau ^{\prime} )$ are two statistical functions of the atom and can be explicitly given by

10 $${({C}_{ij}^{A})}_{b}(\tau ,\tau ^{\prime} )=\frac{1}{2}\langle b|\{{{\rm{r}}}_{i}^{f}(\tau ),{{\rm{r}}}_{j}^{f}(\tau ^{\prime} )\}|b\rangle =\frac{1}{2}\sum _{d}[\langle b|{{\rm{r}}}_{i}^{f}(0)|d\rangle \langle d|{{\rm{r}}}_{j}^{f}(0)|b\rangle {e}^{i{\omega }_{bd}(\tau -\tau ^{\prime} )}+\langle b|{{\rm{r}}}_{j}^{f}(0)|d\rangle \langle d|{{\rm{r}}}_{i}^{f}(0)|b\rangle {e}^{-i{\omega }_{bd}(\tau -\tau ^{\prime} )}],$$

11 $${({\chi }_{ij}^{A})}_{b}(\tau ,\tau ^{\prime} )=\frac{1}{2}\langle b|[{{\rm{r}}}_{i}^{f}(\tau ),{{\rm{r}}}_{j}^{f}(\tau ^{\prime} )]|b\rangle =\frac{1}{2}\sum _{d}[\langle b|{{\rm{r}}}_{i}^{f}(0)|d\rangle \langle d|{{\rm{r}}}_{j}^{f}(0)|b\rangle {e}^{i{\omega }_{bd}(\tau -\tau ^{\prime} )}-\,\langle b|{{\rm{r}}}_{j}^{f}(0)|d\rangle \langle d|{{\rm{r}}}_{i}^{f}(0)|b\rangle {e}^{-i{\omega }_{bd}(\tau -\tau ^{\prime} )}],$$

where *ω*_*bd*_ = *ω*_*b*_ − *ω*_*d*_ and the sum spreads over a complete set of stationary atomic states. The superscript “*f* ” of operators will be omitted hereafter.

### Data availability

All data generated or analysed during this study are included in this published article.

## Results

### Rate of energy change for a static atom near a conducting plate

By employing the previously developed formalism, we now investigate in detail the atomic radiative properties for the case when the surrounding field is confined by a perfectly conducting plane boundary. We assume that an infinite conducting plate is placed perpendicular to the cosmic string at *z* = 0 in space and in its vicinity the atom is placed at rest. Due to the symmetry, we only need to consider the region *z* > 0. In the cylindrical coordinate we use, the atomic trajectory is denoted by *x*(*τ*) = (*τ*, *ρ*_0_, *θ*_0_, *z*_0_). First, the statistical functions (8) and (9) of the electromagnetic field in cosmic string spacetime are indispensable. Obviously, the two functions can be derived from the field’s correlation function

12 $${G}_{ij}(x,x^{\prime} )=\langle 0|{{\rm{E}}}_{i}(x){{\rm{E}}}_{j}(x^{\prime} )|0\rangle =\sum _{\alpha }{{\rm{E}}}_{\alpha i}(x){{\rm{E}}}_{\alpha j}^{\ast }(x^{\prime} ),$$

where $\{{{\rm{E}}}_{\alpha i}(x),{{\rm{E}}}_{\alpha i}^{\ast }(x)\}$ is a complete set of mode functions for the electric field, with the collective index *α* specifying the modes. According to the previous work^47^, the corresponding mode functions for the electric field, meeting the boundary condition (**n** × **E**)|_*z* = 0_ = 0, with **n** being the normal vector to the conducting plate, can be expressed as

13 $${{\rm{E}}}_{\alpha i}^{(\lambda )}(x)={N}_{\alpha }{{\rm{E}}}_{\alpha i}^{(\lambda )}(\rho ,z){e}^{i(\nu m\theta -\omega t)},$$

where its components are explicitly given by

14 $$\begin{array}{ccc}{{\rm{E}}}_{\alpha 1}^{(0)}(\rho ,z) & = & -k\gamma {J^{\prime} }_{|\nu m|}(\gamma \rho )\sin (kz),\\ {{\rm{E}}}_{\alpha 2}^{(0)}(\rho ,z) & = & -ik\nu m{J}_{|\nu m|}(\gamma \rho )\sin (kz),\\ {{\rm{E}}}_{\alpha 3}^{(0)}(\rho ,z) & = & {\gamma }^{2}{J}_{|\nu m|}(\gamma \rho )\cos (kz),\end{array}$$

for the TM modes (*λ* = 0) and

15 $$\begin{array}{ccc}{{\rm{E}}}_{\alpha 1}^{(1)}(\rho ,z) & = & -\omega \frac{\nu m}{\rho }{J}_{|\nu m|}(\gamma \rho )\sin (kz),\\ {{\rm{E}}}_{\alpha 2}^{(1)}(\rho ,z) & = & -i\omega \gamma \rho {J^{\prime} }_{|\nu m|}(\gamma \rho )\sin (kz),\\ {{\rm{E}}}_{\alpha 3}^{(1)}(\rho ,z) & = & 0,\end{array}$$

for the TE modes (*λ* = 1). Here *α* denotes collectively the quantum numbers (*λ*, *γ*, *k*, *m*), *N*_*α*_ is the normalization coefficient, $\omega =\sqrt{{\gamma }^{2}+{k}^{2}}$, *J*_*n*_(*x*) is the Bessel function and the prime denotes the derivative operation. In the region *z* > 0, the mode functions can be normalized as

16 $${\int }_{0}^{\infty }d\rho \rho {\int }_{0}^{\frac{2\pi }{\nu }}d\theta {\int }_{0}^{\infty }dz\,{{\bf{E}}}_{\alpha }^{(\lambda )}\cdot {{\bf{E}}}_{\alpha ^{\prime} }^{(\lambda ^{\prime} )\ast }=\frac{\omega }{2}{\delta }_{\alpha \alpha ^{\prime} },$$

and accordingly we obtain

17 $${N}_{\alpha }^{2}=\frac{\nu }{2{\pi }^{2}\gamma \omega },$$

for both TM and TE modes. Substituting the above mode functions into equation (12), the non-zero components of the field’s correlation function along the atomic trajectory are found to be

18 $${G}_{11}(x(\tau ),x({\tau }^{{\rm{^{\prime} }}}))=\sum _{m=-{\rm{\infty }}}^{{\rm{\infty }}}{\int }_{0}^{{\rm{\infty }}}d\gamma {\int }_{0}^{{\rm{\infty }}}dk\,\frac{\nu }{2{\pi }^{2}\gamma \omega }\,({k}^{2}{\gamma }^{2}{J^{\prime} }_{|\nu m|}^{2}(\gamma {\rho }_{0})+{\omega }^{2}\frac{{\nu }^{2}{m}^{2}}{{\rho }_{0}^{2}}{J}_{|\nu m|}^{2}(\gamma {\rho }_{0})){\sin }^{2}(k{z}_{0}){e}^{-i\omega (\tau -\tau ^{\prime} )},$$

19 $${G}_{22}(x(\tau ),x({\tau }^{{\rm{^{\prime} }}}))=\sum _{m=-{\rm{\infty }}}^{{\rm{\infty }}}{\int }_{0}^{{\rm{\infty }}}d\gamma {\int }_{0}^{{\rm{\infty }}}dk\,\frac{\nu }{2{\pi }^{2}\gamma \omega }({k}^{2}{\nu }^{2}{m}^{2}{J}_{|\nu m|}^{2}(\gamma {\rho }_{0})+{\omega }^{2}{\gamma }^{2}{\rho }_{0}^{2}{J^{\prime} }_{|\nu m|}^{2}(\gamma {\rho }_{0})){\sin }^{2}(k{z}_{0}){e}^{-i\omega (\tau -\tau ^{\prime} )},$$

20 $$\begin{array}{ccc}{G}_{33}(x(\tau ),x(\tau ^{\prime} )) & = & \sum _{m=-{\rm{\infty }}}^{{\rm{\infty }}}{\int }_{0}^{{\rm{\infty }}}d\gamma {\int }_{0}^{{\rm{\infty }}}dk\,\frac{\nu }{2{\pi }^{2}\omega }{\gamma }^{3}{J}_{|\nu m|}^{2}(\gamma {\rho }_{0}){\cos }^{2}(k{z}_{0}){e}^{-i\omega (\tau -\tau ^{\prime} )},\end{array}$$

for *i* = *j* and

21 $${G}_{13}(x(\tau ),x(\tau ^{\prime} ))={G}_{31}(x(\tau ),x(\tau ^{\prime} ))=\sum _{m=-{\rm{\infty }}}^{{\rm{\infty }}}{\int }_{0}^{{\rm{\infty }}}d\gamma {\int }_{0}^{{\rm{\infty }}}dk(-\frac{\nu }{2{\pi }^{2}\omega })k{\gamma }^{2}{J^{\prime} }_{|\nu m|}(\gamma {\rho }_{0}){J}_{|\nu m|}(\gamma {\rho }_{0})\,\sin (k{z}_{0})\cos (k{z}_{0}){e}^{-i\omega (\tau -\tau ^{\prime} )},$$

for *i* ≠ *j*. Notably, the last correlation functions (equation (21)) prove to be nonvanishing and this is in a sharp contrast with the corresponding result in a free cosmic string spacetime^49^. Insert equations (18–21) and equation (11) into expression (6), assume Δ*τ* = *τ* − *τ*_0_ → ∞, then we can obtain the contribution of vacuum fluctuations to the rate of change of the atomic energy,

22 $${\langle \frac{d{H}_{A}(\tau )}{d\tau }\rangle }_{{\rm{v}}{\rm{f}}}=-\,\frac{{e}^{2}}{6\pi }\sum _{d}{\omega }_{bd}^{4}({\rm{\Theta }}({\omega }_{bd})-{\rm{\Theta }}(-{\omega }_{bd}))\,[{|\langle b|{{\rm{r}}}_{i}(0)|d\rangle |}^{2}{f}_{i}(|{\mathop{\rho }\limits^{ \sim }}_{0}|,|{\mathop{z}\limits^{ \sim }}_{0}|,\nu )+2{\rm{R}}{\rm{e}}(\langle b|{{\rm{r}}}_{1}(0)|d\rangle \langle d|{{\rm{r}}}_{3}(0)|b\rangle )g(|{\mathop{\rho }\limits^{ \sim }}_{0}|,|{\mathop{z}\limits^{ \sim }}_{0}|,\nu )],$$

in which Θ(*ω*_*bd*_) is the standard step function, Re refers to the real part of a complex number. In addition, we have defined the functions

23 $$\begin{array}{ccc}{f}_{1}({\mathop{\rho }\limits^{ \sim }}_{0},{\mathop{z}\limits^{ \sim }}_{0},\nu ) & = & \frac{3\nu }{4}\sum _{m=-{\rm{\infty }}}^{{\rm{\infty }}}{\int }_{0}^{1}d\sigma [1\,-\,\cos \,(2{\mathop{z}\limits^{ \sim }}_{0}\sqrt{1-{\sigma }^{2}})]\frac{\sigma }{\sqrt{1-{\sigma }^{2}}}[(2-{\sigma }^{2}){J}_{|\nu m+1|}^{2}({\mathop{\rho }\limits^{ \sim }}_{0}\sigma )\\  &  & +\,{\sigma }^{2}{J}_{|\nu m|+1}({\mathop{\rho }\limits^{ \sim }}_{0}\sigma ){J}_{|\nu m|-1}({\mathop{\rho }\limits^{ \sim }}_{0}\sigma )],\end{array}$$

24 $$\begin{array}{ccc}{f}_{2}({\mathop{\rho }\limits^{ \sim }}_{0},{\mathop{z}\limits^{ \sim }}_{0},\nu ) & = & \frac{3\nu }{4}\sum _{m=-{\rm{\infty }}}^{{\rm{\infty }}}{\int }_{0}^{1}d\sigma [1\,-\,\cos \,(2{\mathop{z}\limits^{ \sim }}_{0}\sqrt{1-{\sigma }^{2}})]\frac{\sigma }{\sqrt{1-{\sigma }^{2}}}[(2-{\sigma }^{2}){J}_{|\nu m+1|}^{2}({\mathop{\rho }\limits^{ \sim }}_{0}\sigma )\\  &  & -\,{\sigma }^{2}{J}_{|\nu m|+1}({\mathop{\rho }\limits^{ \sim }}_{0}\sigma ){J}_{|\nu m|-1}({\mathop{\rho }\limits^{ \sim }}_{0}\sigma )],\end{array}$$

25 $${f}_{3}({\tilde{\rho }}_{0},{\tilde{z}}_{0},\nu )=\frac{3\nu }{2}\sum _{m=-\infty }^{\infty }{\int }_{0}^{1}d\sigma [1+\,\cos \,\mathrm{(2}{\tilde{z}}_{0}\sqrt{1-{\sigma }^{2}})]\frac{{\sigma }^{3}}{\sqrt{1-{\sigma }^{2}}}{J}_{|\nu m|}^{2}({\tilde{\rho }}_{0}\sigma ),$$

26 $$g({\mathop{\rho }\limits^{ \sim }}_{0},{\mathop{z}\limits^{ \sim }}_{0},\nu )=-\,\frac{3\nu }{4}\sum _{m=-{\rm{\infty }}}^{{\rm{\infty }}}{\int }_{0}^{1}d\sigma \,\sin (2{\mathop{z}\limits^{ \sim }}_{0}\sqrt{1-{\sigma }^{2}}){\sigma }^{2}{J}_{|\nu m|}({\mathop{\rho }\limits^{ \sim }}_{0}\sigma )[{J}_{|\nu m|-1}({\mathop{\rho }\limits^{ \sim }}_{0}\sigma )-{J}_{|\nu m|+1}({\mathop{\rho }\limits^{ \sim }}_{0}\sigma )],$$

where ${\tilde{\rho }}_{0}$ = *ω*_*bd*_*ρ*_0_ and ${\tilde{z}}_{0}$ = *ω*_*bd*_*z*_0_. In order to obtain the above results, we have utilized the recurrence relations of the Bessel function^73^,

27 $${J}_{n-1}(x)+{J}_{n+1}(x)=\frac{2n}{x}{J}_{n}(x),\,{J}_{n-1}(x)-{J}_{n+1}(x)=2{J^{\prime} }_{n}(x),$$

and the identical relation

28 $$\sum _{m=-{\rm{\infty }}}^{{\rm{\infty }}}({J}_{|\nu m|+1}^{2}(x)+{J}_{|\nu m|-1}^{2}(x))=2\sum _{m=-{\rm{\infty }}}^{{\rm{\infty }}}{J}_{|\nu m+1|}^{2}(x).\,(\nu \ge 1)$$

Likewise, substituting equations (18–21) and equation (10) into expression (7), the contribution of radiation reaction is given by

29 $$\begin{array}{ccc}{\langle \frac{d{H}_{A}(\tau )}{d\tau }\rangle }_{{\rm{r}}{\rm{r}}} & = & -\,\frac{{e}^{2}}{6\pi }\sum _{d}{\omega }_{bd}^{4}({\rm{\Theta }}({\omega }_{bd})+{\rm{\Theta }}(-{\omega }_{bd}))[{|\langle b|{{\rm{r}}}_{i}(0)|d\rangle |}^{2}{f}_{i}(|{\mathop{\rho }\limits^{ \sim }}_{0}|,|{\mathop{z}\limits^{ \sim }}_{0}|,\nu )\\  &  & +2{\rm{R}}{\rm{e}}(\langle b|{{\rm{r}}}_{1}(0)|d\rangle \langle d|{{\rm{r}}}_{3}(0)|b\rangle )(g(|{\mathop{\rho }\limits^{ \sim }}_{0}|,|{\mathop{z}\limits^{ \sim }}_{0}|,\nu )].\end{array}$$

By comparison with the result for a static atom in a free cosmic string spacetime^49^, we can assert that the presence of the conducting plate affects both vacuum fluctuations and radiation reaction contributions to the rate of change of the atomic energy, as they both are related to the atom-plate separation. Remarkably, we find that, in addition to the modification of the oscillating factor $\cos \,\mathrm{(2}{\tilde{z}}_{0}\sqrt{1-{\sigma }^{2}})$ in the functions *f*_*i*_(${\tilde{\rho }}_{0}$, ${\tilde{z}}_{0}$, *ν*), there appears an extra cross contribution in our result, i.e., the term containing the function *g*(${\tilde{\rho }}_{0}$, ${\tilde{z}}_{0}$, *ν*). According to the atom-field coupling (4), it can be sure that this stems from the existence of the components (21) of the electric field correlation function.

A sum of equations (22) and (29) gives the total contribution

30 $${\langle \frac{d{H}_{A}(\tau )}{d\tau }\rangle }_{{\rm{t}}{\rm{o}}{\rm{t}}}=-\frac{{e}^{2}}{3\pi }\sum _{{\omega }_{d} < {\omega }_{b}}{\omega }_{bd}^{4}[{|\langle b|{{\rm{r}}}_{i}(0)|d\rangle |}^{2}{f}_{i}({\mathop{\rho }\limits^{ \sim }}_{0},{\mathop{z}\limits^{ \sim }}_{0},\nu )+\,2{\rm{R}}{\rm{e}}(\langle b|{{\rm{r}}}_{1}(0)|d\rangle \langle d|{{\rm{r}}}_{3}(0)|b\rangle )g({\mathop{\rho }\limits^{ \sim }}_{0},{\mathop{z}\limits^{ \sim }}_{0},\nu )].$$

Obviously, only the summands with *ω*_*d*_ < *ω*_*b*_ contribute to the total rate of energy change. This shows that only the atomic spontaneous emission process (*ω*_*bd*_ > 0) is allowed and the spontaneous excitation process (*ω*_*bd*_ < 0) is still forbidden, even if the atom is immersed in the fluctuating field confined by a combination of a conducting plane boundary and a cosmic string geometry. The total rate of energy change is directly related to the atom-string distance, the atom-plate separation and the extend of the polar angle deficit. Also, we note that the result is contingent on the specific polarization direction of the atom. In particular, for an isotropically polarized atom, the result reduces to

31 $${\langle \frac{d{H}_{A}(\tau )}{d\tau }\rangle }_{{\rm{t}}{\rm{o}}{\rm{t}}}=-\frac{{e}^{2}}{3\pi }\sum _{{\omega }_{d} < {\omega }_{b}}{\omega }_{bd}^{4}{|\langle b|{\bf{r}}(0)|d\rangle |}^{2}[\frac{1}{3}\,[{f}_{1}({\mathop{\rho }\limits^{ \sim }}_{0},{\mathop{z}\limits^{ \sim }}_{0},\nu )+{f}_{2}({\mathop{\rho }\limits^{ \sim }}_{0},{\mathop{z}\limits^{ \sim }}_{0},\nu )+{f}_{3}({\mathop{\rho }\limits^{ \sim }}_{0},{\mathop{z}\limits^{ \sim }}_{0},\nu )]\pm \frac{2}{3}g({\mathop{\rho }\limits^{ \sim }}_{0},{\mathop{z}\limits^{ \sim }}_{0},\nu )],$$

where the positive and minus signs correspond to the two specific polarization directions $\langle b|{{\rm{r}}}_{i}|d\rangle =\frac{1}{\sqrt{3}}\mathrm{(1},1,\mathrm{1)|}{\bf{r}}|$ and $\langle b|{{\rm{r}}}_{i}|d\rangle =\frac{1}{\sqrt{3}}\mathrm{(1},1,-\mathrm{1)|}{\bf{r}}|$, respectively. It is obvious that the existence of the cross term in equation (30) distinguishes the two polarization directions that bring about the same result in the case of a free cosmic spacetime^49^. So we can conclude that the existence of a conducting plate perturbs the distribution of electromagnetic field in a free cosmic string spacetime via the restriction of boundary conditions and the spacetime under consideration has less symmetry, then accordingly the atomic spontaneous emission process is significantly affected. Besides, it is worth mentioning that the obtained result (30) in our model is very similar to the case for two entangled atoms in a symmetric or antisymmetric state aligned along the direction parallel to the string in a free cosmic string spacetime^50^, provided that the interatomic separation is replaced by the distance of the atom with its image with respect to the plane boundary. This character can be ascribed to the mirror image effects of a conducting plane on the correlation functions of electromagnetic field, which was already found in classical electrodynamics^74^. In fact, such similarity also occurs for transition rates of the atoms coupled to the scalar field^64,75^ or the electromagnetic field^70,76^ in a Minkowski spacetime.

### Analysis of results in different situations

In order to fully reveal the radiative properties of the atom, let us analyze the functions *f*_*i*_(${\tilde{\rho }}_{0}$, ${\tilde{z}}_{0}$, *ν*) and *g*(${\tilde{\rho }}_{0}$, ${\tilde{z}}_{0}$, *ν*) in different cases. We first examine if the result of the Minkowski spacetime can be recovered in the absence of the string (*ν* = 1). By virtue of the addition theorem of the Bessel function^73^

32 $$\sum _{m=-\infty }^{\infty }{J}_{|m|}^{2}(x)=1$$

and the identical relation

33 $$\sum _{m=-\infty }^{\infty }\,{J}_{|m|+1}(x){J}_{|m|-1}(x)=0,\,\sum _{m=-\infty }^{\infty }\,{J}_{|m|}(x)({J}_{|m|-1}(x)-{J}_{|m|+1}(x))=0,$$

we have

34 $${f}_{1}({\tilde{\rho }}_{0},{\tilde{z}}_{0},\,\mathrm{1)}={f}_{2}({\tilde{\rho }}_{0},{\tilde{z}}_{0},\,\mathrm{1)}=1-\frac{3}{8{\tilde{z}}_{0}^{2}}\,\cos \,\mathrm{(2}{\tilde{z}}_{0})+\frac{3-12{\tilde{z}}_{0}^{2}}{16{\tilde{z}}_{0}^{3}}\,\sin \,\mathrm{(2}{\tilde{z}}_{0}),$$

35 $${f}_{3}({\tilde{\rho }}_{0},{\tilde{z}}_{0},\,\mathrm{1)}=1-\frac{3}{4{\tilde{z}}_{0}^{2}}\,\cos \,\mathrm{(2}{\tilde{z}}_{0})+\frac{3}{8{\tilde{z}}_{0}^{3}}\,\sin \,\mathrm{(2}{\tilde{z}}_{0}),$$

36 $$g({\tilde{\rho }}_{0},{\tilde{z}}_{0},\,\mathrm{1)}=0.$$

Then the total rate of energy change reduces to

37 $${\langle \frac{d{H}_{A}(\tau )}{d\tau }\rangle }_{{\rm{t}}{\rm{o}}{\rm{t}}}=-\,\frac{{e}^{2}}{3\pi }\sum _{{\omega }_{d} < {\omega }_{b}}\,{\omega }_{bd}^{4}|\langle b|{{\rm{r}}}_{i}(0)|d\rangle {|}^{2}{f}_{i}({\mathop{\rho }\limits^{ \sim }}_{0},{\mathop{z}\limits^{ \sim }}_{0},1),$$

which is exactly the result for a static atom near a conducting plane boundary in Minkowski spacetime^70^.

When the atom is close enough to the cosmic string, i.e., ${\rho }_{0}\ll {\omega }_{bd}^{-1}$ holds for an arbitrary energy gap *ω*_*bd*_ between two levels of the atom, we have

38 $$\begin{array}{ccc}{f}_{1}({\mathop{\rho }\limits^{ \sim }}_{0},{\mathop{z}\limits^{ \sim }}_{0},\nu ) & \,\approx  & {f}_{2}({\mathop{\rho }\limits^{ \sim }}_{0},{\mathop{z}\limits^{ \sim }}_{0},\nu )\\  & \,\approx  & (\frac{3\nu (\nu +1)}{2(2\nu +1){\rm{\Gamma }}[2\nu ]}-\frac{3\sqrt{\pi }\nu }{{2}^{2\nu +1}{\rm{\Gamma }}[\nu ]{\mathop{z}\limits^{ \sim }}_{0}^{\nu +\frac{1}{2}}}\\  &  & \,\times [(\nu +1){J}_{\nu +\frac{1}{2}}(2{\mathop{z}\limits^{ \sim }}_{0})-2{\mathop{z}\limits^{ \sim }}_{0}{J}_{\nu +\frac{3}{2}}(2{\mathop{z}\limits^{ \sim }}_{0})]){\mathop{\rho }\limits^{ \sim }}_{0}^{2(\nu -1)},\end{array}$$

39 $${f}_{3}({\mathop{\rho }\limits^{ \sim }}_{0},{\mathop{z}\limits^{ \sim }}_{0},\nu )\approx \nu (1-\frac{3}{4{\mathop{z}\limits^{ \sim }}_{0}^{2}}\,\cos \,(2{\mathop{z}\limits^{ \sim }}_{0})+\frac{3}{8{\mathop{z}\limits^{ \sim }}_{0}^{3}}\,\sin \,(2{\mathop{z}\limits^{ \sim }}_{0})),$$

40 $$\begin{array}{ccc}g({\mathop{\rho }\limits^{ \sim }}_{0},{\mathop{z}\limits^{ \sim }}_{0},\nu ) & \,\approx  & -\frac{3\nu [6{\mathop{z}\limits^{ \sim }}_{0}\,\cos \,(2{\mathop{z}\limits^{ \sim }}_{0})+(4{\mathop{z}\limits^{ \sim }}_{0}^{2}-3)\,\sin \,(2{\mathop{z}\limits^{ \sim }}_{0})]}{32{\mathop{z}\limits^{ \sim }}_{0}^{4}}{\mathop{\rho }\limits^{ \sim }}_{0}\\  &  & -\,\frac{3\sqrt{\pi }\nu {\mathop{z}\limits^{ \sim }}_{0}}{{2}^{2\nu +1}{\rm{\Gamma }}[\nu ]{\rm{\Gamma }}[\nu +\frac{5}{2}]}{}_{0}{F}_{1}[\nu +\frac{5}{2};-\,{\mathop{z}\limits^{ \sim }}_{0}^{2}]\,{\mathop{\rho }\limits^{ \sim }}_{0}^{2\nu -1},\end{array}$$

where Γ is the Gamma function and _0_*F*_1_ denotes the Confluent Hypergeometric function. So, for a radially (〈*b*|r_*i*_|*d*〉 = (±1, 0, 0)|**r**|) or tangentially (〈*b*|r_*i*_|*d*〉 = (0, ±1, 0)|**r**|) polarized atom the rate of energy change is proportional to ${\tilde{\rho }}_{0}^{\mathrm{2(}\nu -\mathrm{1)}}$, while for an axially polarized (〈*b*|r_*i*_|*d*〉 = (0, 0, ±1)|***r***|) atom it is proportional to the angle deficit parameter *ν*. So, by comparison with the result (37), we find that the atoms are located in the near zone relative to the string, the decay rate is greatly suppressed for the atoms polarized along the direction perpendicular to the string but is enhanced for the atoms polarized along the direction parallel to the string via the extent of angle deficit. This character is also tenable in the absence of the boundary^49^.

When the atom approaches the conducting plate, i.e., ${z}_{0}\ll {\omega }_{bd}^{-1}$, we have

41 $${f}_{1}({\mathop{\rho }\limits^{ \sim }}_{0},{\mathop{z}\limits^{ \sim }}_{0},\nu )\approx (\frac{3\nu }{2}\sum _{m=-{\rm{\infty }}}^{{\rm{\infty }}}{\int }_{0}^{1}d\sigma \sigma \sqrt{1-{\sigma }^{2}}[(2-{\sigma }^{2}){J}_{|\nu m+1|}^{2}({\mathop{\rho }\limits^{ \sim }}_{0}\sigma )+{\sigma }^{2}{J}_{|\nu m|+1}({\mathop{\rho }\limits^{ \sim }}_{0}\sigma ){J}_{|\nu m|-1}({\mathop{\rho }\limits^{ \sim }}_{0}\sigma )]){\mathop{z}\limits^{ \sim }}_{0}^{2},$$

42 $${f}_{2}({\mathop{\rho }\limits^{ \sim }}_{0},{\mathop{z}\limits^{ \sim }}_{0},\nu )\approx (\frac{3\nu }{2}\sum _{m=-{\rm{\infty }}}^{{\rm{\infty }}}{\int }_{0}^{1}d\sigma \sigma \sqrt{1-{\sigma }^{2}}[(2-{\sigma }^{2}){J}_{|\nu m+1|}^{2}({\mathop{\rho }\limits^{ \sim }}_{0}\sigma ){\sigma }^{2}{J}_{|\nu m|+1}({\mathop{\rho }\limits^{ \sim }}_{0}\sigma ){J}_{|\nu m|-1}({\mathop{\rho }\limits^{ \sim }}_{0}\sigma )]){\mathop{z}\limits^{ \sim }}_{0}^{2},$$

43 $${f}_{3}({\mathop{\rho }\limits^{ \sim }}_{0},{\mathop{z}\limits^{ \sim }}_{0},\nu )\approx (3\nu \sum _{m=-{\rm{\infty }}}^{{\rm{\infty }}}\,{\int }_{0}^{1}d\sigma \frac{{\sigma }^{3}}{\sqrt{1-{\sigma }^{2}}}{J}_{|\nu m|}^{2}({\mathop{\rho }\limits^{ \sim }}_{0}\sigma ))-(3\nu \sum _{m=-{\rm{\infty }}}^{{\rm{\infty }}}\,{\int }_{0}^{1}d\sigma {\sigma }^{3}\sqrt{1-{\sigma }^{2}}{J}_{|\nu m|}^{2}({\mathop{\rho }\limits^{ \sim }}_{0}\sigma )){\mathop{z}\limits^{ \sim }}_{0}^{2},$$

44 $$g({\mathop{\rho }\limits^{ \sim }}_{0},{\mathop{z}\limits^{ \sim }}_{0},\nu )\approx (-\frac{3\nu }{2}\sum _{m=-{\rm{\infty }}}^{{\rm{\infty }}}\,{\int }_{0}^{1}d\sigma \sqrt{1-{\sigma }^{2}}{\sigma }^{2}{J}_{|\nu m|}({\mathop{\rho }\limits^{ \sim }}_{0}\sigma )[{J}_{|\nu m|-1}({\mathop{\rho }\limits^{ \sim }}_{0}\sigma )-{J}_{|\nu m|+1}({\mathop{\rho }\limits^{ \sim }}_{0}\sigma )]){\mathop{z}\limits^{ \sim }}_{0}.$$

So the rate of energy change for a radially or tangentially polarized atom is proportional to ${\tilde{z}}_{0}^{2}$, while for an axially polarized atom it is nearly doubled compared with the case in the absence of the boundary^49^. This indicates that when the atoms are located in the near zone relative to the boundary, the decay rate is always suppressed for the atoms polarizable parallel to the boundary but is enhanced for the atoms polarizable perpendicular to the boundary. We note that the behavior of decay rate for an atom near the string is very similar to that near the boundary, especially when *ν* = 2. By the way, when the atom is far from the conducting plate (${z}_{0}\gg {\omega }_{bd}^{-1}$), due to the rapidly oscillating factors $\cos \,\mathrm{(2}{\tilde{z}}_{0}\sqrt{1-{\sigma }^{2}})$ and $\sin \,\mathrm{(2}{\tilde{z}}_{0}\sqrt{1-{\sigma }^{2}})$ in the integrals equations (23–26), the boundary-induced modification terms can be neglected, then accordingly the result for a static atom in a free cosmic string spacetime is exactly recovered.

**Figure 1:**
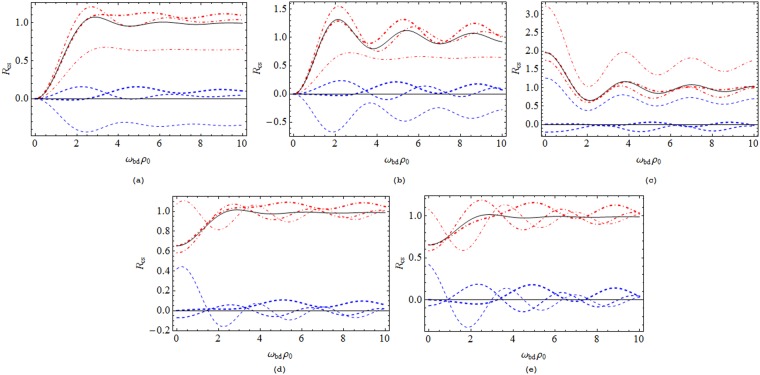
The total decay rate (dot-dashed lines) and the boundary-induced contribution (dashed lines) for a static atom near a conducting plate in the cosmic string spacetime (*ν* = 2), as a function of the atom-string distance. The thin, medium and thick lines correspond to certain atom-plate separations *ω*_*bd*_*z*_0_ = 1, 3, 5, respectively. As a measuring standard, the solid curves show the corresponding results in a free cosmic string spacetime. The decay rates are depicted in the units of that of a static atom in a free Minkowski spacetime (${e}^{2}{\omega }_{bd}^{3}|{\bf{r}}{|}^{2}\mathrm{/3}\pi$). (**a**) The case of radial polarization, (**b**) The case of tangential polarization, (**c**) The case of axial polarization, (**d**) The case of “positive-going” isotropic polarization, (**e**) The case of “negative-going” isotropic polarization.

**Figure 2:**
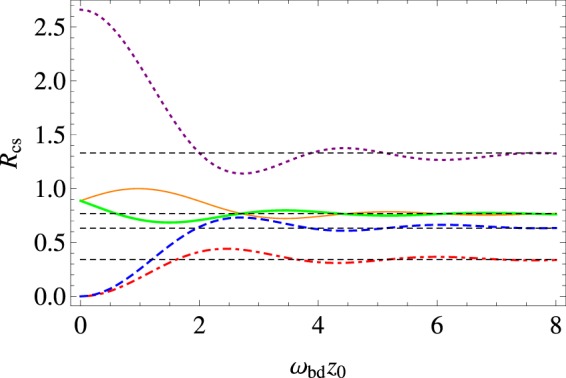
Decay rate of a static atom near a conducting plate in the cosmic string spacetime (*ν* = 2), as a function of the atom-plate separation. The atom-string distance is fixed as *ω*_*bd*_*ρ*_0_ = 1. The dotdashed, dashed, dotted, thin solid and thick solid lines refer to the cases of radial, tangential, axial, “positive-going” isotropic and “negative-going” isotropic polarizations, respectively. The transverse dashed lines refer to their corresponding results in a free cosmic string spacetime. The decay rates are depicted in the units of that of a static atom in a free Minkowski spacetime (${e}^{2}{\omega }_{bd}^{3}|{\bf{r}}{|}^{2}\mathrm{/3}\pi$).

When the atom is located on the general position, we give some numerical results of the atomic decay rate from the initial state |*b*〉 to the final state |*d*〉. For a good display effect, we choose the parameter *ν* = 2 and consider some specific polarization cases. In Fig. 1, the behaviors of the total and the boundary-induced decay rates are depicted as a function of the atom-string distance with certain atom-plate separations. In general, the total decay rate and the boundary-induced contribution both oscillate with the increase of the distance of the atom with the string. Obviously, the modification effects of the boundary rely on the position of the atom relative to the string. Specifically, the boundary-induced modification value can be positive or negative, depending on the atom-plate separation and the atom-string distance. This signifies that the existence of the conducting plane boundary can either enhance or weaken the decay rate of the atom in a free cosmic string spacetime. Especially for an atom close to the string, the behavior of the decay rate varies markedly with the polarization cases. In the case of isotropic polarization, the extent of the modification contribution due to the boundary is relatively concentrated and small as compared with other polarization cases. By comparing the subfigures 1d and 1e, we learn that the existence of the boundary clearly distinguishes the“positive-going” ($\langle b|{{\rm{r}}}_{i}|d\rangle =\frac{1}{\sqrt{3}}\mathrm{(1},1,\mathrm{1)|}{\bf{r}}|$) and “negative-going” ($\langle b|{{\rm{r}}}_{i}|d\rangle =\frac{1}{\sqrt{3}}\mathrm{(1},1,-\mathrm{1)|}{\bf{r}}|$) isotropic polarizations. Furthermore, by Fig. 2, we give the behavior of the decay rate with the variation of the atom-plate separation with a fixed atom-string distance as *ω*_*bd*_*ρ*_0_ = 1. We find that with the increase of the atom-plate separation, the decay rates oscillate around their corresponding results in a free cosmic string spacetime and the amplitudes gradually decrease. For an atom close to the plate, the behavior of the decay rate is also diverse for different polarization cases. Let us notice that here the numerical results are in line with our preceding analytical analysis.

## Discussion

We have investigated the rate of energy change for a static multi-level atom near an infinite, perfectly conducting plane boundary in the background of a cosmic string. It is found that by using DDC formalism, the existence of the boundary modifies both vacuum fluctuations and radiation reaction contributions of the quantum electromagnetic field to the atomic spontaneous emission rate and the spontaneous excitation process is still forbidden by the interplay between the two contributions. Our results show that the total decay rate and the boundary-induced contribution both depend on the atom-string distance, the atom-plane separation, the extend of planar angle deficit induced by the string, and the polarization direction of the atom with respect to the string and the boundary. With the variation of these parameters, the boundary-induced contribution is either positive or negative, and thus can enhance or weaken the decay rate. In particular, when the atom is close to the plane boundary (${z}_{0} < {\omega }_{bd}^{-1}$), the decay rate is greatly restrained for an atom polarized in the direction parallel to the boundary, while for an atom polarized in the direction perpendicular to the boundary it is nearly doubled, as compared with the case in the absence of the boundary. Notably, due to the existence of the boundary, there appears an extra cross term contribution in our result. The existence of the cross term can distinguish certain polarization directions. For instance, for a general polarized atom, the decay rate in our model is different for the two specific polarization directions $\langle b|{{\rm{r}}}_{i}|d\rangle =1/\sqrt{|a{|}^{2}+|b{|}^{2}+|c{|}^{2}}(a,b,c)|{\bf{r}}|$ and $\langle b|{{\rm{r}}}_{i}|d\rangle =1/\sqrt{|a{|}^{2}+|b{|}^{2}+|c{|}^{2}}(a,b,-c)|{\bf{r}}|$, in a sharp contrast with the case in a free cosmic string spacetime. This distinction can be comprehensible in physics, as the presence of the boundary significantly perturbs the distribution of the electric field in a free cosmic string spacetime, the spacetime under consideration has less symmetry, and accordingly the radiative properties of atoms coupled to the field change. By means of the model of a combination of a conducting plane boundary and a cosmic string geometry, theoretically the regulation and control to the transition processes of atoms becomes more flexible as the atomic transition rates depend on more parameters, as compared with the case of a free cosmic string spacetime^49^ or that of a Minkowski spacetime with a conducting plate^70^. In future work, we can change the geometric configuration of boundaries. For instance, we consider the presence of a coaxial conducting cylindrical shell in the cosmic string spacetime and then study the effects of the cylindrical boundary on the transition rates of an atom inside or outside the shell.
